# Identification of KLK10 as a therapeutic target to reverse trastuzumab resistance in breast cancer

**DOI:** 10.18632/oncotarget.13104

**Published:** 2016-11-04

**Authors:** Zhuo Wang, Beihong Ruan, Yi Jin, Yulong Zhang, Jiaqiu Li, Liyuan Zhu, Wenxia Xu, Lifeng Feng, Hongchuan Jin, Xian Wang

**Affiliations:** ^1^ Department of Medical Oncology, Key lab of Biotherapy in Zhejiang, Sir Run Run Shaw Hospital, Medical School of Zhejiang University, Hangzhou, China; ^2^ Laboratory of Cancer Biology, Key lab of Biotherapy in Zhejiang, Sir Run Run Shaw Hospital, Medical School of Zhejiang University, Hangzhou, China; ^3^ Department of Clinical Medicine, Ningbo University, Ningbo, China

**Keywords:** trastuzumab resistance, KLK10, breast cancer, HER2

## Abstract

Trastuzumab, the first antibody widely used in anti-HER2 targeted therapy, dramatically improved the overall outcome of HER2 positive breast cancer patients. However, trastuzumab resistance emerged as a major problem in its clinical application. In order to explore mechanisms underlying trastuzumab resistance, we performed RNA-Seq to analyze the gene expression variation in trastuzumab resistant breast cancer cell line. The sequencing result was then combined with the relevant data in TCGA database to conduct a co-expression analysis. We found a series of differentially expressed genes with potential contributions to trastuzumab resistance. Among them, KLK10 was verified to be a potential therapeutic target for reversing trastuzumab resistance. In summary, this study provides a new clue to screen molecular targets and predictive biomarkers for trastuzumab resistance.

## INTRODUCTION

Breast cancer accounts for the highest morbidity and mortality among all cancers in female globally [1]. Nearly 30% breast cancer patients have HER2 gene amplification [2]. Trastuzumab, the first antibody widely used in anti-HER2 targeted therapy, dramatically improved the overall survival of HER2 positive breast cancer patients. Recently, it has been approved to treat HER2 positive gastric cancer [3]. With its extensive application, trastuzumab resistance emerged as a major problem. Novel targets are expected to reverse trastuzumab resistance. Unfortunately, no effective targets or biomarkers have been approved for trastuzumab resistance. Most of such efforts to identify biomarkers or targets for trastuzumab resistance were based on the molecular mechanism of trastuzumab [4, 5]. More practical alternative approaches would be necessary to identify biomarkers to predict and targets to reverse trastuzumab resistance.

RNA-Seq(RNA sequencing) technology has been commonly used in high throughput analysis of genome-wide gene expression [6]. In addition, the Cancer Genome Atlas (TCGA) project collects high throughput analyses such as gene expression profiling, exon sequencing, SNP genotyping, genomic DNA methylation profiling and microRNA profiling along with clinical data of each patient [7]. In this study, we are trying to combine our RNA-Seq analysis of trastuzumab resistant breast cancer cells with TCGA database to discover potential biomarkers and therapeutic targets for trastuzumab resistance in breast cancer.

## RESULTS

### Establishment of trastuzumab resistant breast cancer cell line

**Figure 1:**
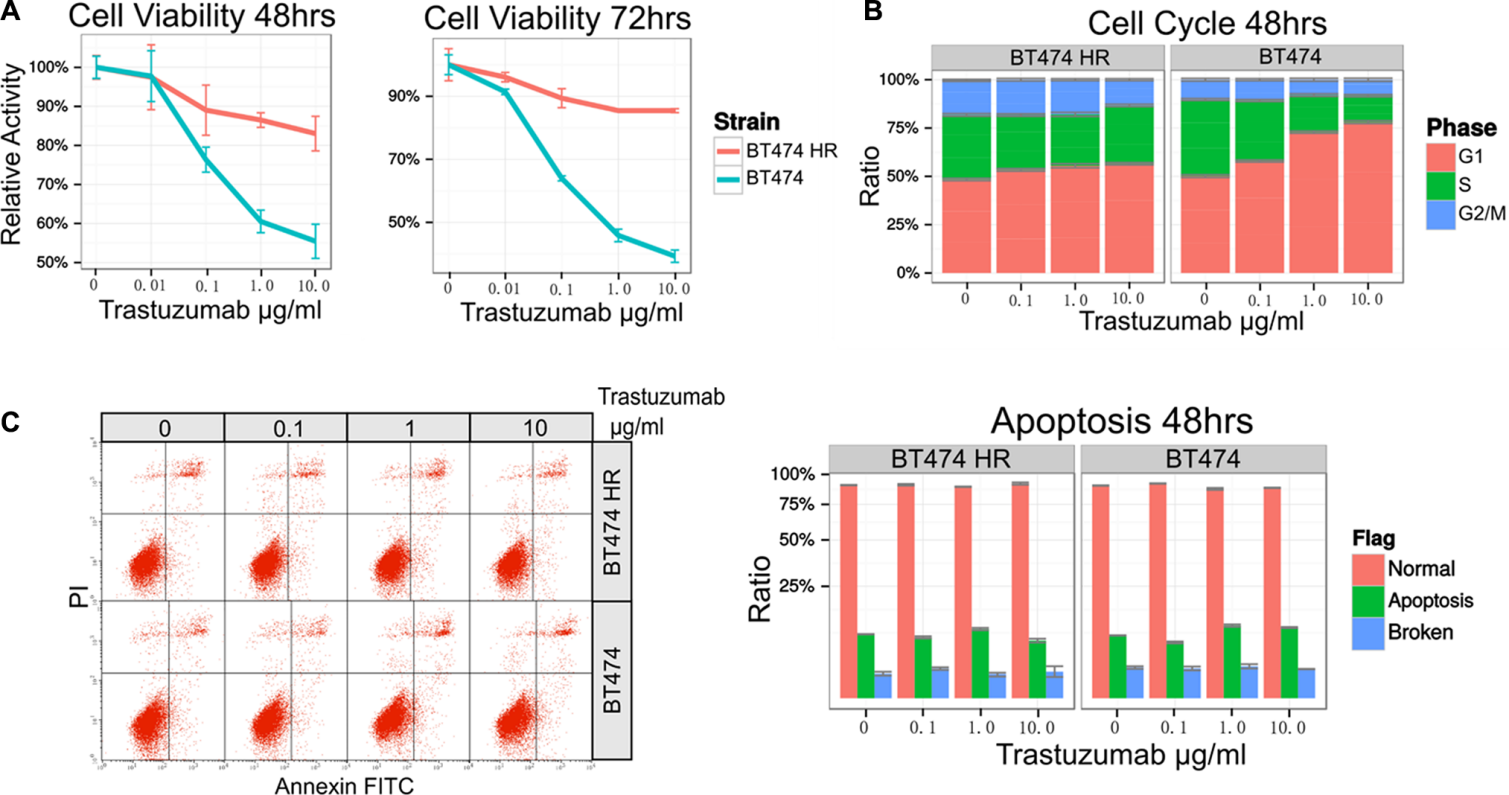
Establishment of Trastuzumab resistant breast cancer cell line (**A**) Cell viability in the presence of various concentration of trastuzumab were determined by MTS assay. The cell cycle distribution (**B**) and cell apoptosis (**C**) were determined by flow cytometry analysis.

BT474 HR (Herceptin Resistant) cells were established by culturing BT474 cells with 1μg/ml Trastuzumab for 6 months and 4 μg/ml Trastuzumab for 3 months. No obvious cellular morphology changes were observed in BT474 and BT474 HR. As expected, trastuzumab could remarkably inhibit the growth of BT474 but not BT474 HR cells (Figure 1A). To determine why trastuzumab can inhibit cell growth, cell apoptosis and cell cycle distribution were determined after trastuzumab treatment. Significant changes were observed in the distribution of cell cycle phases in BT474 after trastuzumab treatment (Figure 1B). Trastuzumab could induce G1 phase arrest strikingly in BT474 cells in a dose-dependent manner, but not in BT474 HR. However, Trastuzumab failed to induce apoptosis in neither BT474 nor BT474 HR (Figure 1C and 1D).

### RNA expression profiling of BT474 and BT474HR cells

**Figure 2:**
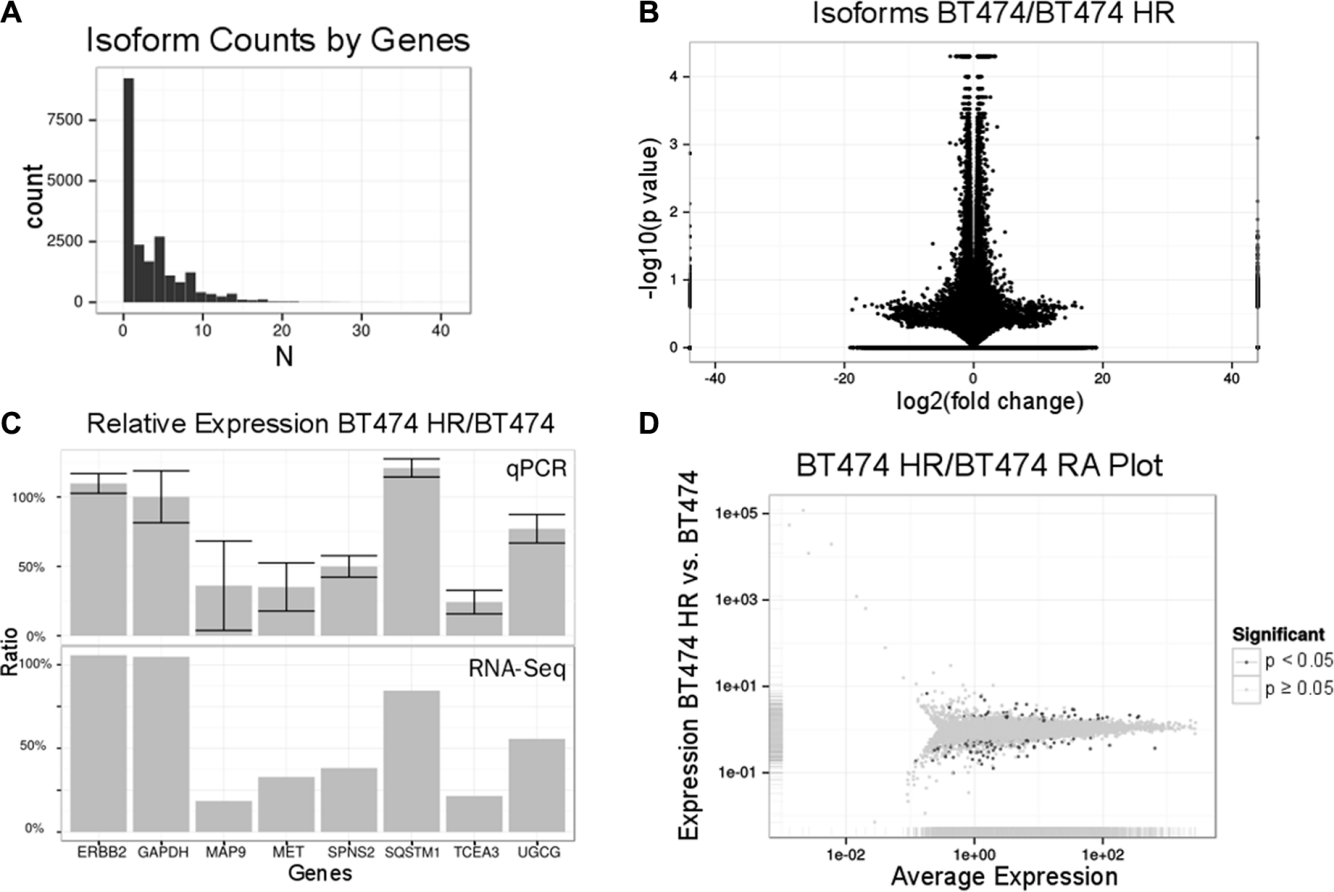
RNA expression profiling of BT474 HR cells The distribution of transcripts counts per gene from RNA-Seq analysis was shown in (**A**). The X axis represented the number of transcripts per gene and the Y axis represented transcripts count number. Statistical significance versus fold-change distribution of differential expression of BT 474/BT474 HR was shown in (**B**). (**C**), RNA-Seq results was verified by quantitative real-time PCR (upper panel). The result of RNA-Seq were shown in the lower panel. GAPDH, ERBB2 and SQSTM1 were used as the control. Relative expression levels and the average expression levels were shown in (**D**). The X axis represented the average expression and the Y axis represented the fold-change of expression of BT474 HR/BT474. Statistically significant (*p* < 0.05) transcripts are highlighted.

We used RNA-Seq to reveal changes of transcriptome in BT474 and BT474 HR cells. 65,677 differentially expressed transcripts from 16,170 genes were received through RNA-Seq analysis. Genes had a mean transcript variant number of about 3 (1–41) (Figure 2A). The volcano plot was used to observe for abnormal signals (Figure 2B). After filtering out these outliers, 246 genes were found to be statistical significance (*p* < 0.05). Next, quantitative real-time PCR was used to validate differentially expressed genes including MAP9, MET, SPNS2, TCEA3 and UGCG using highly and equally expressed GAPDH, ERBB2 and SQSTM1 as the control. For all these tested genes, the expression determined by quantitative real-time PCR was consistent with RNA-Seq results (Figure 2C). The representative transcripts for each protein coding gene, which had a higher level of expression, were selected for further analysis. The data was plotted with expression ratio vs. average expression (Figure 2D), and there was neither obvious skewed distribution nor abnormal signal after filtering. Finally, differential expression data of 12,228 transcripts was extracted as representatives of effective protein coding genes.

### Co-expression analysis

**Figure 3:**
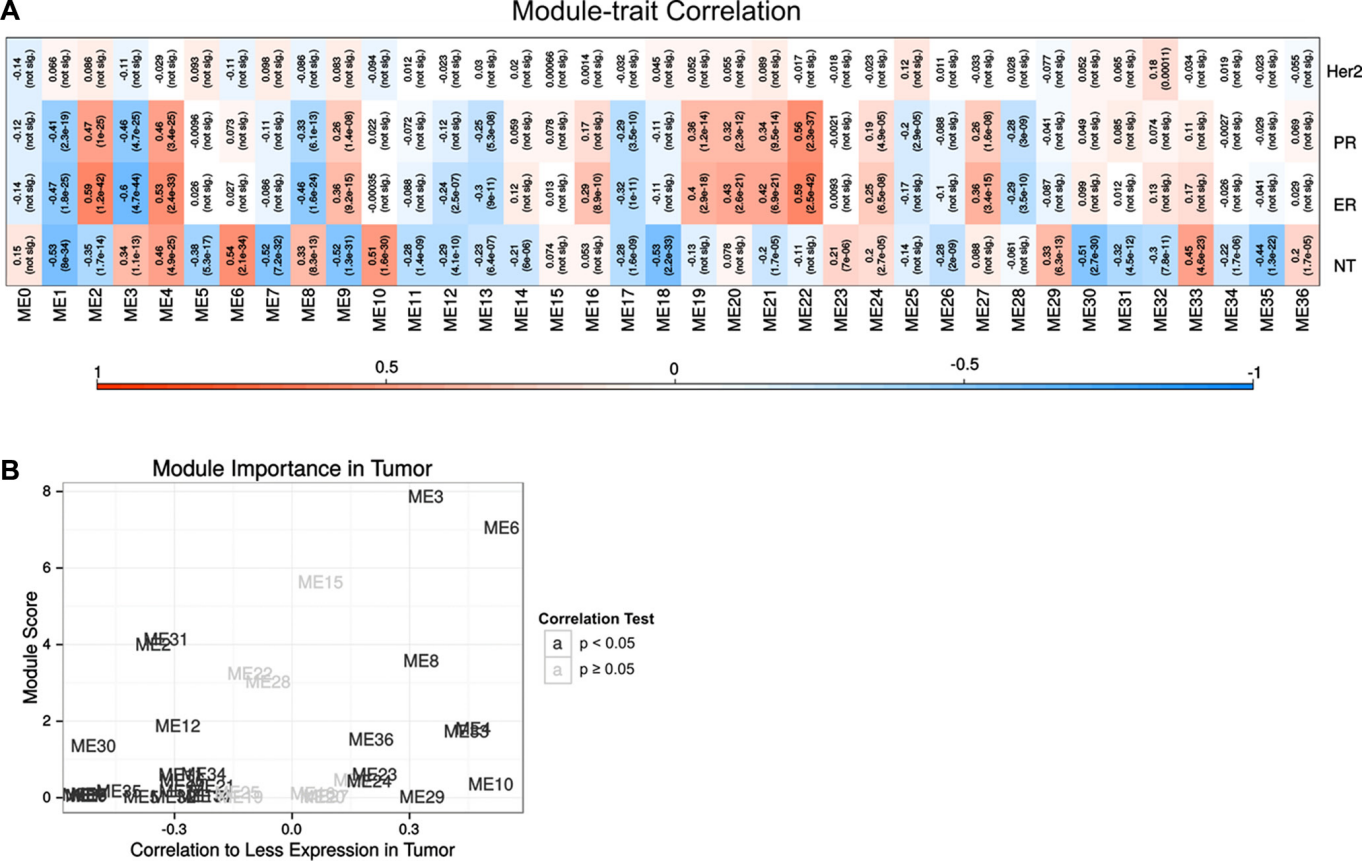
Co-expression analysis of RNA-Seq and TCGA database (**A**) The correlation between co-expression cluster's eigengene and whether the tissue type (normal tissue or tumor), ER, PR and HER2 states. In each module, there were two rows, the first row was correlation. −1 represented negative correlation and 1 represented positive correlation. The second row was *p* value, “not sig” meant no significant. (**B**) The top 10% differentially expressed genes enriched in each clusters. The X axis represented the correlation to tumor or normal tissue, and the Y axis was –ln(p) from bionmial test, represented the likelihood to trastuzumab resistance.

To explore functions of differential genes systematically, gene co-expression network was utilized. In this method, we selected genes both meaningful in our RNA-Seq data and in expression profile from TCGA. A total of 9,913 genes were obtained in two data sets. In TCGA, 444 cases were in accordance with the co-expression analysis criteria. This data set was analyzed by WGCNA clustering and 36 gene sets were finally clustered. The clusters were then correlated with expression features in tumor tissues, ER, PR and HER2 states (Figure 3A). For summarizing such clusters, the principal component of each cluster or module eigengene (ME) was used. For instance, ME0 had no significant correlations with all features, while HER2 status had no significant correlation to any clusters but ME32. Different cluster had various degrees of relevance to tissue types, ER and PR. Highly similar correlation patterns of ER and PR implied the clustering of co-expression was a good indicator for biological functions.

If drug resistance-related genes were irrelevant to co-expression cluster genes, selected genes that changed most remarkably in the expression should be uniformly distributed in the co-expression cluster gene sets. In contrast, the relationship between this gene set and drug resistance was significant when a particularly large number of differentially expressed genes were presented in some co-expression gene sets. Therefore, the top 10% differentially expressed genes were selected, and the distributions of their frequency of occurrence in the co-expression gene cluster sets were compared and statistically tested to show whether they consisted more than 10% of a gene set. As shown in Figure 3B, ME3 and ME6 gene sets had more top 10% differentially expressed genes. It implied that these gene sets were more significantly related with drug resistance. Also, they were more related to tumor, representing good sources for targets and biomarkers identification.

### Target validation

Therefore, KLK10 from ME3 and KLK11 from ME6 were selected as potential targets for further validations. Receptor tyrosine kinase encoding EPHA3 from ME4, which had a low score, was chosen as a control.

**Figure 4:**
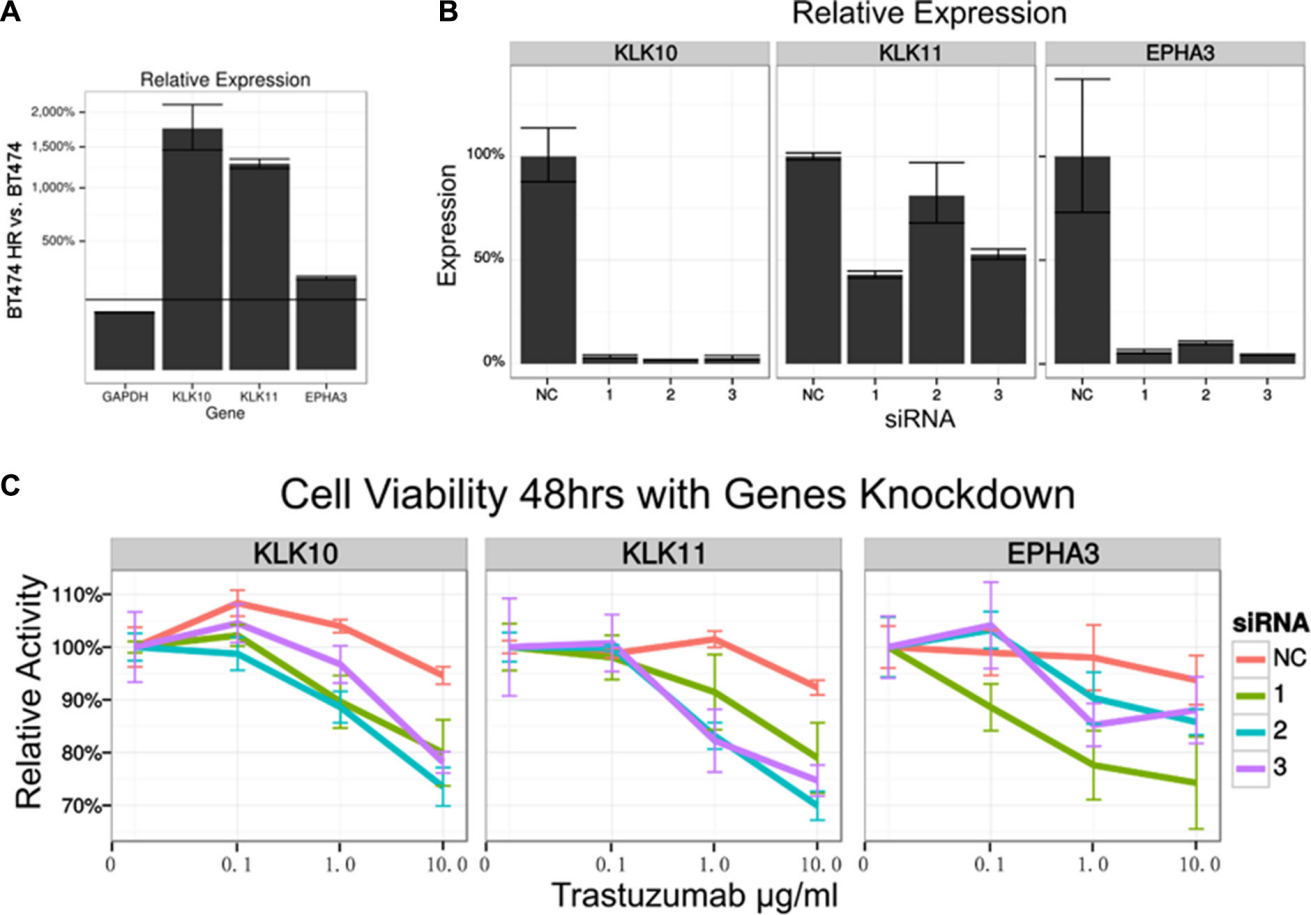
Functional validation of potential targets (**A**) Quantitative real-time PCR was performed to compare KLK10, KLK11 and EPHA3 mRNA expression in BT474 HR and BT474. (**B**) The mRNA expression was determined by quantitative real-time PCR after transiently transfected with NC siRNA or KLK10, KLK11 and EPHA3 targeting siRNA in BT474 HR. (**C**) The cells viability was determined by MTS assay after trastuzumab treatment for 48 hours in BT474 HR.

Quantitative real-time PCR validated the differential expression of these genes (Figure 4A). To further explore the biological relevance of these genes to drug resistance, trastuzumab induced growth inhibition before and after knock-down of these potential targets were determined by MTS assay. While depletion of EPHA3 or KLK11 had no significant effects on trastuzumab sensitivity (*p* > 0.05), KLK10 knock-down significantly reversed resistance to trastuzumab (*p* < 0.05) (Figure 4B and 4C).

**Figure 5:**
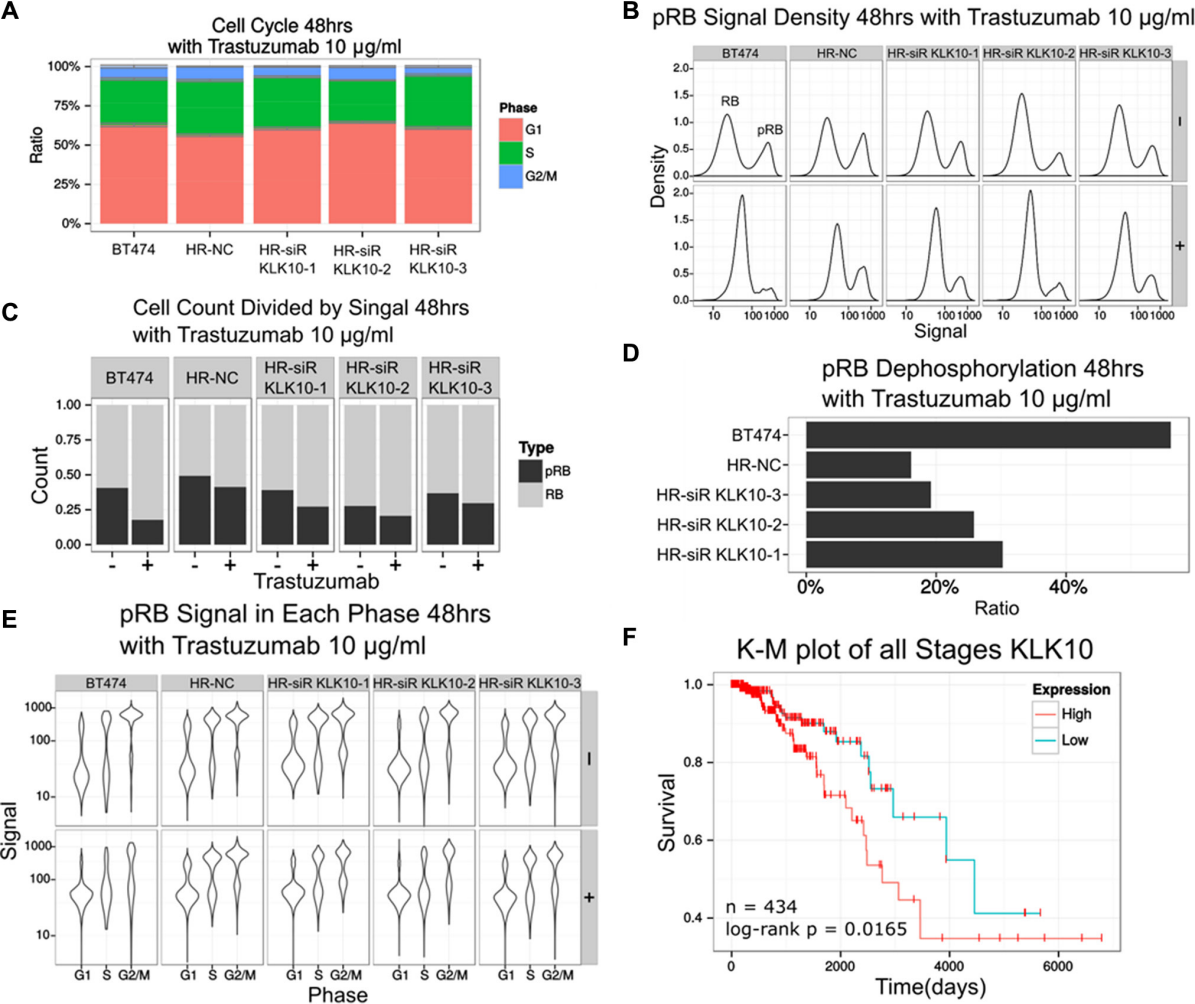
KLK10 is a potential target to reverse trastuzumab resistance (**A**) Cell cycle distribution of trastuzumab and KLK10 siRNA treated cells were determined by flow cytometry. (**B**) pRB signal density in various cells as indicated were analyzed by flow cytometry. (**C**) Numbers of cells with RB or pRB signal were counted by flow cytometry. (**D**) Ratios of pRB dephosphorylation were calculated based on the detection of pRB by flow cytometry. (**E**) The distribution of pRB signal in different cell cycle phases in cells treated as indicated were analyzed by flow cytometry. (**F**) Effect of KLK10 expression on overall survival of HER2 positive breast cancer patients were determined by Kaplan-Meier plot. Log-rank test was used for statistical analysis.

To further confirm the relevance of KLK10 to trastuzumab resistance, cell cycle distribution was measured in the presence of trastuzumab and KLK10 depletion. After KLK10 depletion, trastuzumab successfully induced G1 arrest (Figure 5A). As dephosphorylated Retinoblastoma (RB) protein is a well-known marker for G1 phase [8], we detected phosphorylation level of RB in BT474 HR cells treated with trastuzumab and KLK10 siRNA. pRB in BT474 but not BT474 HR cells were indeed decreased after trastuzumab treatment (Figure 5B). Once KLK10 was depleted, trastuzumab successfully decreased pRB in BT474 HR cells (Figure 5B and 5C). Trastuzumab treatment led to almost 60% inhibition of pRB in BT474 cells, but only 17% in BT474 HR cells (Figure 5D). However, KLK10 siRNA significantly increased trastuzumab-induced inhibition of pRB. Furthermore, SFIT method [9] was used to distinguish the cell cycle phases. After knock-down of KLK10, trastuzumab-treated cells were accumulated in G1 phase with low pRB levels (Figure 5E). Together, KLK10 siRNA succeeded to reverse trastuzumab resistance.

Given the significant effect of drug resistance on clinical outcome, we explored the relevance of KLK10 expression to the overall survival of breast cancer patients. According to the median expression of KLK10, 434 patients from TCGA database were divided into high expression group and low expression group (Figure 5F). High expression of KLK10 was related to poor prognosis in HER2 positive breast cancer patients (*n* = 434, log-rank *p* = 0.0165418). Cox proportional hazards regression analysis showed that high expression of KLK10 led to an elevation of 18.1% hazard risk (95% CI:1.015–1.375, *p* = 0.031).

## DISCUSSION

Despite the successful application of trastuzumab for the treatment of HER2 positive breast cancer, its acquired or intrinsic resistance hurdled improvement of breast cancer patients. In this study, we profiled gene expression in a newly established trastuzumab resistant breast cancer model and combined it with publically available database to identify potential targets to reverse trastuzumab resistance. Indeed, KLK10 was increased in trastuzumab-resistant cells and its depletion succeeded to reverse trastuzumab resistance. Importantly, high KLK10 expression was associated with poor prognosis of breast cancer, indicating that KLK10 is a potential target and prognosis predictor for trastuzumab resistance.

Although there are 246 genes detected to be statistically significant differentially expressed between BT474 and BT474 HR cells, some may have no relevance to trastuzumab resistance per se. Therefore, other approaches are needed to screen potential targets for further functional analyses. Co-expression is models of multiple gene expression pattern associated with certain potential biological functions. Co-expression can determine the functional expression pattern of breast tissue while differential expression analysis can reflect the single gene expression patterns in two cell straits, with both random patterns and meaningful patterns related to trastuzumab resistance. By constructing a reasonable score, a combination of both methods can be achieved to get a list of genes with important functions. By enrichment analysis of clustering gene sets and differential expression data, we obtained ME3, ME6 and other gene sets potentially related with trastuzumab resistance. The persistent differentially expressed genes in such gene sets would have much higher chance to be prediction biomarkers and intervention targets for trastuzumab resistance. Indeed, we have successfully identified KLK10 from ME3 as a relevant target to reverse trastuzumab resistance.

The members of KLK family are exocrine protein readily to be detected in the serum. Interestingly, KLK3 is a tumor-associated biomarker well known as prostate specific antigen (PSA). KLK4, KLK5, KLK6 and KLK7 have been found related to the prognosis of ovarian cancer [10–13]. Therefore, it would be interesting to explore whether plasma level of KLK10 are associated with drug resistance and overall survival in breast cancer. In fact, KLK10 also has been found related to the prognosis of breast cancer due to its association with tamoxifen resistance [14]. In summary, by co-expression analysis of TCGA data with gene expression profile of trastuzumab resistant breast cancer, we identified KLK10 as a potential biomarker and intervention target for trastuzumab resistance. We found a series of differentially expressed genes with potential contributions to trastuzumab resistance. Among them, KLK10 was verified to be a potential therapeutic target for reversing trastuzumab resistance.

## MATERIALS AND METHODS

### Cell lines

Human breast cancer cell line BT474 was obtained from American Type Culture Collection (ATCC, Manassas, VA, USA) and maintained in RPIM 1640 supplemented with 10% FBS at 37°C in a humidified, 5% CO_2_ incubator. BT474 HR (Herceptin Resistant) cells were maintained in the presence of 10 μg/ml trastuzumab.

### Cell viability assay

MTS assay was performed with the CellTiter 96^®^ Aqueous No-Radioactive Cell Proliferation Assay Kit (Promega). The cells were transferred into a 96-well plate and cultured overnight before adding trastuzumab or phosphate buffer saline (PBS). 48 h or 72 h later, the cell viability was measured following the manufacturer's instruction. Samples were prepared in triplicates at least.

### RNA extraction and quantitative real-time RT-PCR

Total RNA was extracted by TRIzol reagent (Invitrogen) according to the manufacturer's. RNA concentrations were quantified by NanoDrop™ 2000 (Thermo Scientific). Reverse transcription reaction was performed using 2 μg of total RNA with Quantscript RT Kit (Tiangen biotechnology, Beijing, China). The mRNA expression level was determined by quantitative real-time PCR using Bestar^®^ SybrGreen qPCR mastermix (DBI) and LightCycler 480^®^ II Real-Time PCR System (Roche). Primers used are listed in Table 1.

**Table 1:** Primers used in the study

Gene	Forward (5′–3′)	Reverse (5′–3′)
GAPDH	GGAGTCAACGGATTTGGT	GTGATGGGATTTCCATTGAT
ERBB2	AGGAGTGCGTGGAGGAAT	CCAGATGGGCATGTAGGAG
MAP9	CAATTACAGCTCGCTCAG	CTTTGGTTATGTTACCGTTT
MET	ATTGATTGCTGGTGTTGT	TTTCTGTAGTTGGGCTTAC
SPNS2	TGGACAGGTACACCGTGGCA	GGGAATGAAGGAGCTGGAGA
SQSTM1	AGAACGTTGGGGAGAGTGTG	GCGATCTTCCTCATCTGCTC
TCEA3	CAAGTCTTCTGCCTCCTCC	AATCATCGTCCGCCTTCA
UGCG	TGATCCAGCCATTGATGT	CCGTGAACCAAGCCTACT
KLK10	CTTGGACCCCGAAGCCTATG	CACAGTGGCTTGTTTCCGC
KLK11	TCTCACAGCAGCCAAGGAAC	CAGAGTAGCCGCGTCTTCTC
EPHA3	GAGGTCAAATACTATGAAAAGCAGG	TGTTCGTCCCATATCCAGCG

### RNA-seq

RNA-Seq was performed with Ion Total RNA-Seq kit v2 of Ion Proton™ (Ion Torrent, Life Technologies). GRCh37 reference genome from the phase 3 of the 1000 Genomes Project was used for RNA-Seq alignment. Gene annotation of GRCh37.p13 GENCODE Release 19 was utilized to determine the splicing site annotation files. STAR v2.4.1d was used [15] and 2-pass strategy preliminary comparison was adopted, annotations splicing site information was utilized, and the parameters were same in ENCODE Projec [16]. To align the unsuccessful sequence of STAR, Bowtie2 v2.2.4 was used with a more sensitive parameter “—local —very-sensitive-local” [17]. Finally, Samtools v1.1 was utilized to combine the results of the two methods.

### Obtain the differential expression by annotation database

The numbers of transcript fragments per kilobase of per million mapped reads (FPKM) were calculated to determine the relative amount of mRNA in the cells. Data was normalized with the default FPKM method of cuffdiff v2.2.1 from Cufflink [18]. GRCh37.p13 GENCODE Release 19 was selected for annotation and cummeRbund on the platform R v3.2.4 was used to determine the differential expression according to the comparison of BT474 and BT474 HR data.

### TCGA database extraction

All data which included both Level 3 microarray data and clinical data in XML format data set of breast cancer patients by TCGA DATA Portal were selected. XML package of R was used to parse clinical data in XML format. The required information was extracted and merged into the corresponding microarray data. Microarray data downloaded from UNC AgilentG4502 platform including 593 cases of specimens. These specimens were from 529 breast cancer patients' tumor and normal tissues. The patients' clinical profiles including the age, gender, race, follow-up times (days), end event, the method of first time confirmed diagnosis, histological type, ER and PR status, immunohistochemistry and FISH of HER2, pathological stage and grade were extracted.

### Co-expression analysis

WGCNA v1.46 of R was used to cluster the dataset [19]. Correlation analyses of other clinical indicators were adopted to identify potential tumor-associated co-expression patterns. For each gene co-expression cluster, the differential expression result was evaluated with enrichment analysis by binomal test and calculated a score for the association of drug resistance.

### siRNA transfection

KLK10, KLK11 and EPHA3 depletion were achieved by transfection with siRNA (Gene pharma, Shanghai, China). Cells were seeded overnight in 6-well plates (4 × 10^5^/well) and transfected with siRNA duplexes (20 nM) using Lipofectamine^TM^ RNAiMAX transfection reagent (Invitrogen) following the manufacturer's instruction. The sequences of various siRNAs are listed in Table 2.

**Table 2:** siRNAs used in the study

Gene	sense (5′–3′)	antisense (5′–3′)
KLK10-#1	UCUUCAACGGCCUCUCGUUTT	AACGAGAGGCCGUUGAAGATT
KLK10-#2	CCCGGAGAGUGAAGUACAATT	UUGUACUUCACUCUCCGGGTT
KLK10-#3	GGUCACCAACAACAUGAUATT	UAUCAUGUUGUUGGUGACCTT
KLK11-#1	GCAGUUAAUCCUGCUUGCUTT	AGCAAGCAGGAUUAACUGCTT
KLK11-#2	GCAACAUCACAGACACCAUTT	AUGGUGUCUGUGAUGUUGCTT
KLK11-#3	GGAGACGAUGAAGAACAAUTT	AUUGUUCUUCAUCGUCUCCTT
EPHA3-#1	GCUCUGUUCUCGACAGCUUTT	AAGCUGUCGAGAACAGAGCTT
EPHA3-#2	CCAGGUUUCUACAAGGCAUTT	AUGCCUUGUAGAAACCUGGTT
EPHA3-#3	GCGGUCAGCAUCACAACUATT	UAGUUGUGAUGCUGACCGCTT

### Flow cytometry analysis

4 × 10^5^ cells cultured overnight in 6-well plates were treated with or without trastuzumab and harvested after 48 or 72 hours. Cell apoptosis were detected with apoptosis kit (FITC Annexin V Apoptosis Detection Kit I, BD Pharmingen^TM^). Briefly, cells were washed twice with cold PBS and incubated in 100 μl binding buffer with 5 μl of FITC Annexin V and 5 μl PI for 15 minutes in the dark. For cell cycle analysis, cells were resuspended in 200 μl cold PBS, and then added into 1 ml 70% ethanol. After an hour, cells were transferred to 450 μl PBS with 40 μl RNAse (Sigma) and 10 μl PI. For pRB expression analysis [20], room-temperature 1.5% (vol/vol) paraformaldehyde was added for 10 minutes to fix the cells washed twice with cold PBS. Fixed cells were permeabilized by slowly adding cold 100% methanol. 100 μl PBS with 1 μg pRB antibody (Phospho-RB Ser807/811, Cell Signaling Technology) was then added to mark the cells. After the incubation of fluorochrome-conjugated secondary antibody, cells were then stained with PI as previously described.

### Survival analysis

434 breast cancer (invasive ductal carcinoma) patients with follow-up information from TCGA DATA Portal were chosen. According to the median of KLK10 expression, patients were divided into high and low expression group, Kaplan-Meier Plot was used to reveal differences in survival between the two groups. The log-rank test was used for the statistical analysis of overall survival. A cox proportional hazards regression analysis was conducted to quantify the risk.
